# Molecular Markers of Antimalarial Drug Resistance in *Plasmodium falciparum* From Kumasi, Ghana, 2023

**DOI:** 10.1111/tmi.70048

**Published:** 2025-10-15

**Authors:** Albert Dennis Kegya, Elizabeth Oppong, Eric Darko, Kwame Ayisi Boateng, Richard Odame Phillips, Melina Heinemann, Michael Ramharter, Clement Igiraneza, Jules Minega Ndoli, Frank P. Mockenhaupt, Welmoed van Loon

**Affiliations:** ^1^ Kumasi Centre for Collaborative Research in Tropical Medicine Kumasi Ghana; ^2^ University Hospital, Kwame Nkrumah University of Science and Technology Kumasi Ghana; ^3^ Center for Tropical Medicine, Bernhard‐Nocht‐Institute for Tropical Medicine & I. Dep. of Medicine University Medical Centre Hamburg‐Eppendorf Hamburg Germany; ^4^ Department of Internal Medicine University Hospital and University of Zurich Zurich Switzerland; ^5^ German Center for Infection Research, Partner Site Hamburg‐Lübeck‐Borstel‐Riems Hamburg Germany; ^6^ University Teaching Hospital of Butare, University of Rwanda Butare Rwanda; ^7^ Institute of International Health, Charité Center for Global Health, Charité – Universitaetsmedizin Berlin Berlin Germany

**Keywords:** artemisinin, Ghana, malaria, *pfaat1*, *pfcoronin*, *pfk13*, *pfmdr1*, resistance

## Abstract

**Objectives:**

Artemisinin partial resistance has emerged in East Africa and is feared to spread across the continent, potentially compromising artemisinin‐based combination treatment. Molecular markers can serve as early warning signals for the development of specific drug resistance. We aimed at updating information on relevant resistance markers among *Plasmodium falciparum* from Kumasi, Ghana, collected in 2023.

**Methods:**

*P. falciparum* isolates were obtained from 200 patients with falciparum malaria. Mutations in *pfk13* (propeller domain), *pfcoronin* (P76S), *pfmdr1* (N86Y, Y184F, Y1246D) and *pfaat1* (S258L) were genotyped. A subset of patients was microscopically examined for the presence of asexual malaria parasites on day‐3 of treatment with artemether‐lumefantrine.

**Results:**

2.2% of isolates exhibited non‐synonymous *pfk13* mutations including the candidate artemisinin partial resistance marker P527H in addition to S522C, C532S and I590V. *Pfcoronin* P76S, previously associated with artemether‐lumefantrine failure in malaria imported from Africa, was present in 29.7% and associated with a lower parasite density. The predominance of the *pfmdr1* pattern N86‐184F‐D1246 (NFD) suggested impaired sensitivity to lumefantrine. *Pfaat1* S258L was common at 64.7%. The resistance markers were not associated with each other, and they were not linked with day‐3 parasite positivity.

**Conclusions:**

While the efficacy of artemisinin‐based combination treatment in Ghana is still high, isolated critical *pfk13* polymorphisms were identified as well as a considerable prevalence of a potentially relevant *pfcoronin* marker, against a background of *pfmdr1* signals for impaired lumefantrine sensitivity. Continuous molecular monitoring is necessary to detect threats to artemisinin‐based combination treatment efficacy at an early stage.

## 1 Background

Partial artemisinin resistance (AR) of the malaria parasite *Plasmodium falciparum* has emerged in East Africa in the mid‐2010s, notably in Uganda, Rwanda, Tanzania and Eritrea [1, 2, 3]. In that region, parasites with mutations conferring AR are increasing and spreading, and further spread across the continent is feared. AR initially manifests as delayed parasite clearance following treatment, for example, with an artemisinin‐based combination therapy (ACT). Due to concurrent or secondary emergence of resistance to the non‐artemisinin companion drug, actual ACT failure may follow after a few years, as has been observed in the Greater Mekong Region, where AR appeared some two decades ago [1, 4]. In Uganda, in vitro activity of dihydroartemisinin and lumefantrine has decreased over time, parallel to the increase of mutant parasites [5], but so far, ACTs in Africa are still efficacious [1, 6, 7]. In this context, early and close monitoring of antimalarial resistance trends is important also beyond the current AR epicentre in East Africa, not lastly to prepare for potential countermeasures or drug policy changes. Molecular markers of antimalarial resistance can serve as early warning signals in this regard.

In Southeast Asia and in East Africa, antimalarial resistance (AR) is associated with mutations in the *P. falciparum Kelch13* (*pfk13*) propeller domain. These mutations are categorized as validated (associated with delayed parasite clearance in patients and in vitro/ex vivo resistance) or candidate (delayed parasite clearance only) [6]. In addition, variants of *pfcoronin* encoding a *P. falciparum* actin‐binding protein have been implicated in AR [8]. In Israeli patients with malaria imported largely from West Africa, the P76S mutation was associated with treatment failure to artemether‐lumefantrine (AL) [9]. *P. falciparum multidrug resistance‐1* gene (*pfmdr1*) variants affect susceptibility to the main non‐artemisinin partner drugs, that is, lumefantrine and amodiaquine. *Pfmdr1* N86 (wildtype) is linked with decreased sensitivity to lumefantrine but with increased sensitivity to amodiaquine, and haplotype N86‐184F‐D1246 (NFD) with decreased artemether‐lumefantrine sensitivity [10, 11]. Moreover, the *P. falciparum putative amino acid transporter* (*pfaat1*) gene, particularly the S258L variant, has recently been suggested as a novel chloroquine‐resistance marker. Gene editing has demonstrated its association with reduced *P. falciparum* susceptibility to lumefantrine and increased susceptibility to amodiaquine, albeit at levels below clinical significance [12].

Ghana adopted ACTs for the treatment of uncomplicated malaria in 2004, with artesunate‐amodiaquine (ASAQ) and AL as first‐line therapies and dihydroartemisinin‐piperaquine as the second‐line option. In the present study, we assessed mutations in *pfk13*, *pfcoronin*, *pfmdr1* and *pfaat1* among malaria patients in Kumasi, south‐central Ghana, as an inventory of current parasite mutations that may affect ACTs in that area and as a baseline for subsequent monitoring.

## 2 Materials and Methods

### 2.1 Study Setting and Patient Recruitment

Kumasi is a city of approximately 400,000 inhabitants, with the metropolitan area covering a population of more than 4 million. Located in south‐central Ghana, a tropical climate with an annual precipitation of 1500 mm prevails. Between June and August 2023, patients attending the University Hospital on the campus of the Kwame Nkrumah University for Science and Technology were screened for malaria by rapid diagnostic test (MERISCREEN Malaria *Pf* HRP‐II Ag, India) and/or microscopy of Giemsa‐stained blood films. Informed written consent was obtained from malaria patients (or guardians in case of minors) prior to recruitment into the study. The study protocol was approved by the Committee on Human Research, Publication and Ethics of the School of Medicine and Dentistry, Kwame Nkrumah University of Science and Technology (CHRPE/AP/454/22). Patients were clinically examined, a brief history was taken, and axillary temperature was measured.

### 2.2 Sample Collection

Venous blood was collected into EDTA, and haemoglobin (Hb) was measured by a CBC analyser (SYSMEX XN‐31, UK). Malaria parasites were counted per 200 white blood cells (WBCs), and parasite density was calculated assuming 8000 WBCs *per* μL. Whole blood was aliquoted and dried on Whatman 3MM paper (Citvia, USA).

### 2.3 DNA Extraction and Molecular Analysis

DNA was extracted from dried blood spots (QIAamp DNA Blood Mini Kit, Qiagen, Hilden, Germany), and *Plasmodium* presence and species were confirmed by nested PCR assays [13]. All samples were subjected to *pfk13* propeller domain PCR [14]. A random selection of 109 *P. falciparum* positive samples was PCR amplified for *pfmdr1* region 1 (codons 61–236) and 49 samples for *pfmdr1* region 2 (codons 1106–1298) [15]. *Pfk13* and *pfmdr1* amplicons were Sanger‐sequenced commercially (EurofinsGenomics, Germany) and aligned to reference genes of 3D7 (PlasmoDB.org) using CodonCode version 9.1. *Pfmdr1* haplotypes were counted for loci 86, 184 and 1246, and only if no mixed alleles were detected. All samples were genotyped for *pfcoronin* S76L and a random selection of 131 samples for *pfaat1* S258L by allele‐specific and cost‐effective high‐resolution melting‐curve assay on a Roche Lightcycler 480 II. Primer and probe sequences, and assay details are presented in Table S1 and Figure S1.

### 2.4 Statistical Analysis

Descriptive analysis included proportions (%) as well as means and medians as appropriate. For parasite density, the geometric mean was computed with respective 95% confidence intervals (CI). Fisher's exact tests and Student's *t*‐test were applied to explore associations between drug resistance markers, medical history, laboratory data and day‐3 positivity. Welch's *t*‐test was used to compare logarithmically transformed parasite densities between markers and linear regression including age to account for confounding.

## 3 Results

Two‐hundred patients with RDT and microscopy confirmed *P. falciparum* malaria were analysed. Median age was 21 years (range, 8–65); 17% (34/200) of patients were below 18 years of age, and males predominated (70.8%, 136/192). The mean axillary temperature at presentation was 37.6°C; 55.1% (109/198) were febrile, and all patients reported a febrile episode in the preceding 2 days. Median Hb was 13.0 g/dL (range, 7.4–16.0; *n* = 143). Antimalarial treatment in the preceding 2 weeks was stated by two (1.0%) patients (i.v. artesunate, 1; AL, 1); these patients were included in further analysis. Geometric mean parasite density (GMPD) was 9770/μL (95% CI, 7929–12,039; *n* = 199). Double species infection with *P. ovale* and *P. malariae* was present in 2.0% (4/200) and 0.5% (1/200), respectively. Approximately half of the patients (109/199; 54.8%) received AL as initial treatment, while the other half were given parenteral artemisinin derivatives (intravenous artesunate, 2.4 mg/kg, at 0, 12, 24 h, continued daily based on the patient's state of consciousness for a maximum of 7 days, or intramuscular artemether, 3.2 mg/kg immediately then 1.6 mg/kg daily for 5 days), each followed by a full course of AL. One in five (18.1%, 36/199) patients was admitted to the hospital. Ninety‐one patients were available for parasitological review on day‐3 of treatment, of whom 10 (11.0%) showed malaria parasites upon microscopy (Table 2).

Molecular markers were successfully typed in 90.0% (180/200) for *pfk13*, 92.5% (185/200) for *pfcoronin* P76S, 92.7% (101/109) for *pfmdr1* N86Y and Y184F, and 81.7% (107/131) for *pfaat1* S258L (Figure S2). As for *pfk13*, four (2.2%) non‐synonymous mutations were detected, including the candidate AR marker P527H (Table 1) as well as six synonymous mutations (2.8%; nucleotide positions 1239 G>T, 1347 T>G, 1407 C>T, 1605 G>A, 1767 C>G, 1881 T>A). *Pfcoronin* P76S was present in almost 30%. All but one isolate exhibited the *pfmdr1* N86 wildtype allele (99.0%), and *pfmdr1* Y184F was seen in 79.2%. All 49 samples sequenced for D1246Y were wildtype. The *pfaat1* S258L variant occurred in almost two‐thirds of isolates (Table 1).

**TABLE 1:** Non‐synonymous mutations in antimalarial resistance markers in Kumasi, Ghana, 2023.

Gene	Variant	Prevalence, *n*/*N* (%)
*Pfk13*	S522C	1/180 (0.6)
	P527H	1/180 (0.6)
	C532S	1/180 (0.6)
	I590V	1/180 (0.6)
*Pfcoronin*	P76S	55/185 (29.7)
*Pfmdr1*	N86 (wildtype)	100/101 (99.0)
	Y184F	80/101 (79.2)
	D1246 (wildtype)	49/49 (100)^a^
	Haplotype NFD^b^	36/45 (80.0)
	Haplotype NYD^b^	9/45 (20.0)
*Pfaat1*	S258L	66/102 (64.7)

^a^ Genotyping not continued because of apparent fixation.

^b^ Haplotypes based on available data for codons N86Y–Y184F–D1246Y, and all samples with mixed‐allele signals excluded.

Positivity for asexual parasites on day‐3 following treatment did not associate with age, sex, GMPD or the individual resistance markers (Table 2). Day‐3 positivity was associated with treatment regimen and occurred significantly more after intramuscular artemether compared with intravenous artesunate or AL alone (OR [95% CI], 5.9 [1.2–32.2], *p* = 0.02). The resistance markers were not associated with each other (Table S2), and they did not show correlation with parasite density, except for *pfcoronin* 76S, where the GMPD (95% CI) was lower (7184/μL [4751–10,864]) compared to infections with *pfcoronin* wildtype parasites (12,794/μL [9954–16,443]; *p* = 0.02). This association remained significant also after adjusting for age in multiple linear regression (*β* [95% CI], 0.2 [0.04–0.5]; *p* = 0.02).

**TABLE 2:** Characteristics of the day‐3 negative and day‐3 positive patients after antimalarial treatment.

	Day‐3 negative	Day‐3 positive	*p*
	*N* = 81	*N* = 10	
Age, median (range), years	21.0 (9.0–52.0)	21.0 (15.0–39.0)	0.7
Female sex, *n*/*N* (%)	25/78 (32.1)	2/10 (20.0)	0.4
Weight, median (range), kg	65.0 (6.0–106.0)	65.0 (48.0–83.0)	0.7
Axillary temperature, median (range), °C	37.6 (35.8–40.9)	37.6 (36.3–40.1)	0.7
GMPD, parasites/μL (95% CI)	8631 (6320‐11,786)	7052 (2735‐18,182)	0.7
Antimalarial treatment			0.02
AL only	49/81 (60.5%)	3/10 (30.0%)	
Intramuscular Artemether, immediately, followed by AL	16/81 (19.8%)	6/10 (60.0%)	
Intra‐venous Artesunate, 3 days, followed by AL	16/81 (19.8%)	1/10 (10.0%)	
Self‐reported pre‐treatment in the 2 weeks preceding initial enrolment	2/81 (2.5%)	0/10 (0%)	0.6
Hospital admission	14/80 (17.5%)	0/10 (0%)	0.2
*Pfk13* any non‐synonymous variant	1/75 (1.3%)	0/6 (0%)	0.8
*Pfmdr1 N84* (wild type)	45/46 (97.8%)	2/2 (100%)	0.8
*Pfmdr1* Y184F (mutant or mixed)	36/46 (78.3%)	1/2 (50%)	0.4
*Pfcoronin P76S* (mutant or mixed)	21/75 (28%)	2/8 (25%)	0.9
*Pfaat1* S258L (mutant or mixed)	30/45 (66.7%)	3/5 (60%)	0.8

## 4 Discussion

Treatment failures of ASAQ or AL in Ghana have remained below the critical WHO threshold of 10% for ACTs so far [7, 16, 17], and in vitro data suggest sensitivity to amodiaquine and lumefantrine, with occasional evidence for reduced artemisinin susceptibility [17, 18]. Polymorphisms of the *pfk13* gene are considered the major molecular markers associated with AR [1, 14]. Globally, more than 250 non‐synonymous mutations in the *pfk13* gene have been reported. However, the majority of these mutations are rare, not linked to resistance or their relevance remains unknown. Some mutations may arise spontaneously, stay unselected and eventually disappear [7, 19, 20]. Moreover, other genetic variants may influence artemisinin susceptibility [1, 8, 9]. For instance, recent data indicate an increasing number of cases of imported malaria in travellers failing to AL treatment despite the absence of *pfk13* mutations [9, 21, 22]. Notwithstanding this limitation, it is reassuring that only 2.2% of the examined parasite isolates exhibited non‐synonymous *pfk13* mutations. However, this includes the candidate marker P527H as well as S522C, which has been reported by WHO as a rare mutation, potentially (due to few cases only) associated with delayed parasite clearance [23]. All *pfk13* variants detected in the present study, that is, S522C, P527H, C532S and I590V, have previously been observed in African *P. falciparum* isolates, all at low prevalence [20, 24, 25]. Specifically, S522C has been found in the Central African Republic, the Democratic Republic of the Congo (DRC), and Gabon, P527H and I590V in Cameroon, and C532S in DRC and Zambia [24, 25, 26]. In Ghana, a decline in wildtype *pfk13* was observed between 2007 and 2016; three quarters of the *pfk13* variants were non‐synonymous, most occurred only once and there was no overlap with the variants observed in the current study [27]. Likewise, in 2012, no validated *pfk13* markers were observed in northern Ghana [28] or in Kumasi [29], and the reported variants do not match the ones seen by us. Malaria samples collected in Ghana in 2018 revealed 16% of non‐synonymous *pfk13* including a low prevalence of the validated markers C580Y and Y493H, common in Southeast Asia. Again, there was no overlap with the variants detected in the present study [30].

*Pfcoronin* variants have been implicated in AR, but findings are not straightforward and possibly depend on the genetic background of the parasite, including *pfk13* [31]. Polymorphisms G50E, R100K and E107V confer reduced artemisinin susceptibility in vitro [8], which reportedly is due to an interference of the mutated protein with haemoglobin endocytosis, eventually affecting artemisinin activation [32]. However, other *pfcoronin* polymorphisms have been linked to in vitro AR in Uganda [33] and to AL failure in imported malaria cases [9]. As for the latter, mutation P76S was found significantly more frequently in patients with imported malaria failing to AL treatment (29%) as compared to successfully treated patients (6%) [9]. Also, P76S reportedly is the most common *pfcoronin* variant in Africa [34] reaching the highest proportion in isolates from West Africa (21%) [35] and 24% in Ghana [36]. We therefore looked into the prevalence of this variant and found it in 30% of isolates. This is roughly twice the figure of 16% observed in *P. falciparum* from Kumasi more than a decade ago [37]. Considering limited sample sizes in both studies, this may reflect a random finding or selection over time. Reduced parasite densities in *P. falciparum* with the *pfcoronin* P76S mutation need to be confirmed in larger samples, in other settings and in vitro, as this might indicate a fitness cost of respective parasites potentially affecting the pace and extent of emergence and spread.

The *pfmdr1* N86 wildtype prevailed in the present study, as did the NFD haplotype. Amodiaquine and lumefantrine exert opposite selective effects on the *pfmdr1* alleles [38, 39] but in Ghana, AL appears to be more commonly used than AQAS [40], matching the observed *pfmdr1* pattern. Most recent data from Central Ghana (2020–2021) show a *pfmdr1* 184F prevalence almost identical to our data, but a tenth of isolates with the mutant 86Y variant [41], which was virtually absent in the present study. Urban–rural differences in antimalarial drug consumption might be involved in that [42]. Unfortunately, there is no reliable marker for lumefantrine sensitivity so far, but the present *pfmdr1* pattern suggests limited sensitivity [10, 11, 38, 39].

*Pfaat1* S258L occurred in two‐thirds of isolates in the present study. This polymorphism has been reported to modulate chloroquine resistance, to slightly reduce in vitro susceptibility to lumefantrine and to increase susceptibility to amodiaquine, to bear a fitness cost and to be co‐selected with a central mutation in the *P. falciparum chloroquine resistance transporter* (*pfcrt* K76T) gene in The Gambia [12]. Similar to a recent study from Rwanda, the prevalence of *pfaat1* S258L in our study was high despite *pfcrt* K76T being rare in Ghana [41]. This argues against a major fitness cost of the *pfaat1* mutation and suggests that predominant AL pressure might select for *pfaat1* S258L. More data on *pfaat1* S258L, including its effects on in vitro and in vivo susceptibilities and fitness, are needed.

A positive malaria blood smear on day‐3 of ACT treatment has been considered a good predictor of subsequent treatment failure [43]. In the present study, this was observed in 11% among a rather small subset of patients for whom these data were available. Treatment with artemether was linked to increased day‐3 positivity. It has previously been reported that intramuscular artemether is inferior to artesunate to treat severe malaria, which could be explained by its variable pharmacokinetics [44]. None of the assessed molecular markers correlated with day‐3 positivity. A major limitation of our study is the lack of long‐term follow‐up in the full patient group to better define the role of the resistance markers in treatment outcome, particularly when considering that mild or beginning AR commonly manifests as late treatment failure [2, 9, 16, 22]. This will be considered in the design of future studies.

In conclusion, among *P. falciparum* isolates obtained from malaria patients in Kumasi, Ghana, in 2023, only a few harboured non‐synonymous *pfk13* mutations. However, that included variants known to affect parasite clearance following artemisinin‐based treatment. Another putative AR marker, *pfcoronin* P76S, was frequent but its role in response to treatment was not discernible in the present study and needs further investigation. The *pfmdr1* pattern suggests impaired sensitivity towards lumefantrine. While the effectiveness of ACTs in Ghana appears to be good at the moment, molecular surveillance and monitoring systems for the early detection of a relevant level of AR resistance markers should be expanded and operated.

## Conflicts of Interest

The authors declare no conflicts of interest.

## Supporting information


**Table S1:** Primers and probes used in PCR assays to assess *Plasmodium* species, to sequence the *pfk13* propeller domain, *pfmdr1* region 1 and 2, and for fluorescence resonance energy transfer (FRET) assays to genotype *pfcoronin* S76L and *pfaat1* S258L.
**Figure S1:** Representative melting curves for fluorescence resonance energy transfer (FRET) assays to genotype *pfcoronin* P76D and *pfaat1* S258L on the Roche LightCycler 480 II. For *pfcoronin* P76S (upper panel), the mutant variant 76S has a higher melting point (65°C) compared to the wild type allele (60°C). For *pfaat1* S258L (lower panel), similarly, the variant 258L has a higher melting point (60°C) compared to the wild type allele (56°C).
**Table S2:** Cross tabulations with frequencies of the assessed antimalarial drug resistance‐associated markers. Categorical variables were analysed using Fisher's exact test, geometric mean parasite densities (GMPD) were compared using Welch's t‐test on log‐transformed values, and respective *p* values were generated.
**Figure S2:** Summary flow chart of sample processing and genotyping success.
