# Supplementary material for: Molecular Markers of Antimalarial Drug Resistance in Plasmodium falciparum From Kumasi, Ghana, 2023
Source: Trop Med Int Health. 2025 Oct 15;31(1):43–8. doi: 10.1111/tmi.70048 (PMC12775893; doi:10.1111/tmi.70048)
Supplement: Supplementary file 1 — Table S1: Primers and probes used in PCR assays to assess Plasmodium species, to sequence the pfk13 propeller domain, pfmdr1 region 1 and 2, and for fluorescence resonance energy transfer (FRET) assays to genotype pfcoronin S76L and pfaat1 S258L. Figure S1: Representative melting curves for fluorescence resonance energy transfer (FRET) assays to genotype pfcoronin P76D and pfaat1 S258L on the Roche LightCycler 480 II. For pfcoronin P76S (upper panel), the mutant variant 76S has a higher melting point (65°C) compared to the wild type allele (60°C). For pfaat1 S258L (lower panel), similarly, the variant 258L has a higher melting point (60°C) compared to the wild type allele (56°C). Table S2: Cross tabulations with frequencies of the assessed antimalarial drug resistance‐associated markers. Categorical variables were analysed using Fisher's exact test, geometric mean parasite densities (GMPD) were compared using Welch's t‐test on log‐transformed values, and respective p values were generated. Figure S2: Summary flow chart of sample processing and genotyping success. [file TMI-31-43-s001.docx]

**Supplementary material**

**Molecular markers of antimalarial drug resistance in *Plasmodium falciparum* from Kumasi, Ghana, 2023**

Albert Dennis Kegya^1^, Elizabeth Oppong^2^, Eric Darko^2^, Kwame Ayisi Boateng^2^, Richard Odame Phillips^1,2^, Melina Heinemann^3,4^, Michael Ramharter^3,5^, Clement Igiraneza^6^, Jules Minega Ndoli^6^, Frank P. Mockenhaupt^7^, Welmoed van Loon^7^*

1. Kumasi Centre for Collaborative Research in Tropical Medicine, Kumasi, Ghana
2. University Hospital, Kwame Nkrumah University of Science and Technology, Kumasi, Ghana
3. Center for Tropical Medicine, Bernhard-Nocht-Institute for Tropical Medicine & I. Dep. of Medicine University Medical Centre Hamburg-Eppendorf, Hamburg, Germany
4. Department of Internal Medicine, University Hospital and University of Zurich, Zurich, Switzerland
5. German Center for Infection Research, Partner Site Hamburg-Lübeck-Borstel-Riems
6. University Teaching Hospital of Butare, University of Rwanda, Butare, Rwanda
7. Institute of International Health, Charité Center for Global Health, Charité – Universitaetsmedizin Berlin, Berlin, Germany

* Corresponding author: Welmoed van Loon, Institute of International Health, Charité Center for Global Health, Charité – Universitätsmedizin Berlin, Augustenburger Platz 1, 13353 Berlin, Germany. E-Mail: [welmoed.van-loon@charite.de](mailto:welmoed.van-loon@charite.de). Phone: +49 30 450 565 764. Fax: +49 30 450 565 989

**Supplementary Table 1.** Primers and probes used in PCR assays to assess *Plasmodium* species, to sequence the *pfk13* propeller domain, *pfmdr1* region 1 and 2, and for fluorescence resonance energy transfer (FRET) assays to genotype *pfcoronin* S76L and *pfaat1* S258L.

| **Assay, primer or probe** | **Sequence (5' to 3')** | **Reference or assay conditions** |
| --- | --- | --- |
| *Plasmodium* spp,  forward primer | TTA AAA TTG TTG CAG TTA AAA CG | [13] |
| *Plasmodium* spp,  reverse primer | CCT GTT GTT GCC TTA AAC TTC | [13] |
| *P. falciparum*, forward primer | TTA AAC TGG TTT GGG AAA ACC AAA TAT ATT | [13] |
| *P. falciparum*, reverse primer | ACA CAA TGA ACT CAA TCA TGA CTA CCC GTC | [13] |
| *P. malariae*, forward primer | ATA ACA TAG TTG TAC GTT AAG AAT AAC CGC | [13] |
| *P. malariae*, reverse primer | AAA ATT CCC ATG CAT AAA AAA TTA TAC AAA | [13] |
| *P. ovale*, forward primer | ATC TCT TTT GCT ATT TTT TAG TAT TGG AGA | [13] |
| *P. ovale*, reverse primer | GGA AAA GGA CAC ATT AAT TGT ATC CTA GTG | [13] |
| *P. vivax*, forward primer | CGC TTC TAG CTT AAT CCA CAT AAC TGA TAC | [13] |
| *P. vivax*, reverse primer | ACT TCC AAG CCG AAG CAA AGA AAG TCC TTA | [13] |
| *pfk13* propeller domain, forward primer | GGG AAT CTG GTG GTA ACA GC | [14] |
| *pfk13* propeller domain, reverse primer | GCC TTG TTG AAA GAA GCA GA | [14] |
| *pfmdr1* region 1, forward primer | TTA AAT GTT TAC CTG CAC AAC ATA GAA AAT T | [15] |
| *pfmdr1* region 1, reverse primer | CTC CAC AAT AAC TTG CAA CAG TTC TTA | [15] |
| *pfmdr1* region 2, forward primer | AAT TTG ATA GAA AAA GCT ATT GAT TAT AA | [15] |
| *pfmdr1* region 2, reverse primer | TAT TTG GTA ATG ATT CGA TAA ATT CAT C | [15] |
| *pfcoronin* S76L, forward primer | GCG CTT TAA AGA TTA ATT CAT TAT TCA T | Designed and provided by TIB MOLBIOL; total reaction volume of 10 µL containing 0.5x Genotype Master (Roche, Hilden), 1.25 mM MgCl_2_, 1.0 µM forward primer, 0.2 µM reverse primer, 0.1 µM SimpleProbe and 1 µL DNA template, in PCR grade H_2_O. Cycling conditions were: initial denaturation for 10 seconds at 95 °C; 45 cycles of 1 second at 95 °C, 1 second at 60 °C, 30 seconds at 72 °C; stepwise annealing with 1 second at 95 °C, 30 seconds at 55 °C, 30 seconds at 45 °C, 30 seconds at 41 °C; melting curve by heating up to 78 °C with a ramp rate of 0.11 °C/seconds. |
| *pfcoronin* S76L, reverse primer | CTC ATG ACG TAT CTC CCA TAT TCT TA |  |
| *pfcoronin* S76L, SimpleProbe | TGA CAA ATC AXI AGG ATG GAA GAT GTA TGA CT –PH  (XI denotes a modified nucleic acid to obtain a Locked Nucleic Acid (LNA), required because of high AT content) |  |
| *pfaat1* S258L, forward primer | TTA CAC CTG GTG GTG TTA GAT C | Probes and primers designed and provided by TIB MOLBIOL; total reaction volume of 10 µL containing 0.5x Genotype Master (Roche, Hilden), 1.5 mM MgCl_2_, 0.5 µM forward primer, 0.1 µM reverse primer, 0.1 µM SimpleProbe and 1 µL DNA template, in PCR grade H_2_O. Cycling conditions were: initial denaturation for 10 seconds at 95 °C; 45 cycles of 1 second at 95 °C, 1 second at 60 °C, 15 seconds at 72 °C; a melting curve starting with 3 seconds at 95 °C, 2 minutes at 40 °C, and heating up to 75 °C with a ramp rate of 1.5. |
| *pfaat1* S258L, reverse primer | GGG AAA TTA AAT ACA TAA AAG ATG GTA C |  |
| *pfaat1* S258L,  SimpleProbe | AAT ATA ACT TAA TAC AAA ACC AAA TGA CA -PH |  |

**Supplementary Figure 1. Representative melting curves for fluorescence resonance energy transfer (FRET) assays to genotype *pfcoronin* P76D and *pfaat1* S258L on the Roche LightCycler 480 II.** For *pfcoronin* P76S (upper panel), the mutant variant 76S has a higher melting point (65 ⁰C) compared to the wild type allele (60 ⁰C). For *pfaat1* S258L (lower panel), similarly, the variant 258L has a higher melting point (60 ⁰C) compared to the wild type allele (56 ⁰C).

**
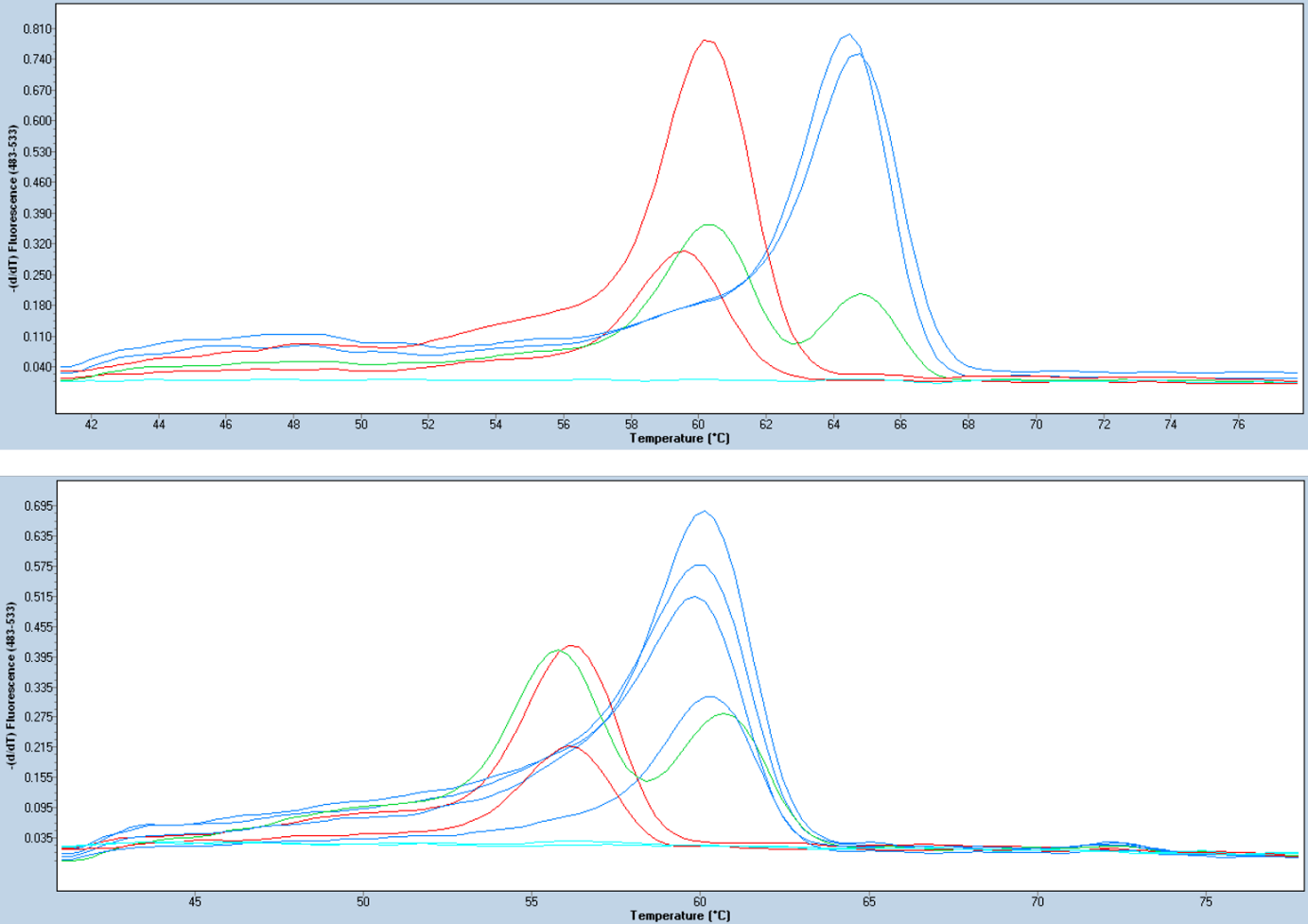
**

**Supplementary Table 2.** Cross tabulations with frequencies of the assessed antimalarial drug resistance-associated markers. Categorical variables were analyzed using Fisher’s exact test, geometric mean parasite densities (GMPD) were compared using Welch’s t-test on log-transformed values, and respective P-values were generated.

|  | ***Pfcoronin* P76S (mutant or mixed)** | ***Pfcoronin* P76**  **(wild type)** | **P-value** |
| --- | --- | --- | --- |
| ***Pfk13*, any non-synonymous variant** | 2/49 (4.1%) | 3/127 (2.4%) | 0.5 |
| ***Pfaat1* 258L**  **(mutant or mixed)** | 19/28 (67.9%) | 48/77 (62.3%) | 0.6 |
| ***Pfmdr1* N84**  **(wild type)** | 31/31 (100%) | 69/70 (98.6%) | 0.5 |
| ***Pfmdr1* Y184F (mutant or mixed)** | 26/31 (83.9%) | 56/72 (77.8%) | 0.5 |
| ***Pfmdr1 D1246Y* (mutant or mixed)** | 18/18 (100%) | 31/31 (100%) | N/A |
| **GMPD (95% CI), parasites/µL** | 7,184 (4,751 – 10,864) | 12,794 (9,954 – 16,443) | 0.02 |
|  | ***Pfaat1 S*258L**  **(mutant or mixed)** | ***Pfaat1 S*258**  **(wild type)** |  |
| ***Pfk13*, any non-synonymous variant** | 2/67 (3%) | 0/36 (0%) | 0.3 |
| ***Pfmdr1* N84**  **(wild type)** | 40/41 (97.6%) | 26/26 (100%) | 0.4 |
| ***Pfmdr1* Y184F (mutant or mixed)** | 34/43 (79.1%) | 19/26 (73.1%) | 0.5 |
| ***Pfmdr1 D1246Y* (mutant or mixed)** | 20/20 (100%) | 20/20 (100%) | N/A |
| **GMPD (95% CI), parasites/µL** | 10,245 (7,124 – 14,734) | 12,883 (8,149 – 20,366) | 0.4 |
|  | ***Pfk13*, any non-synonymous variant** | ***Pfk13* wild type** |  |
| ***Pfaat1* 258L**  **(mutant or mixed)** | 2/2 (100%) | 65/101 (64.4%) | 0.3 |
| ***Pfmdr1* N84**  **(wild type)** | 5/5 (100%) | 95/96 (99%) | 0.8 |
| ***Pfmdr1* Y184F (mutant or mixed)** | 5/5 (100%) | 77/98 (78.6%) | 0.3 |
| ***Pfmdr1 D1246Y* (mutant or mixed)** | N/A | 49/49 (100%) | N/A |
| **GMPD (95% CI), parasites/µL** | 18,642 (2,550 - 13,6241) | 11,079 (8,898 - 13,795) | 0.5 |

**Supplementary Figure 2. Summary flow chart of sample processing and genotyping success.**

**
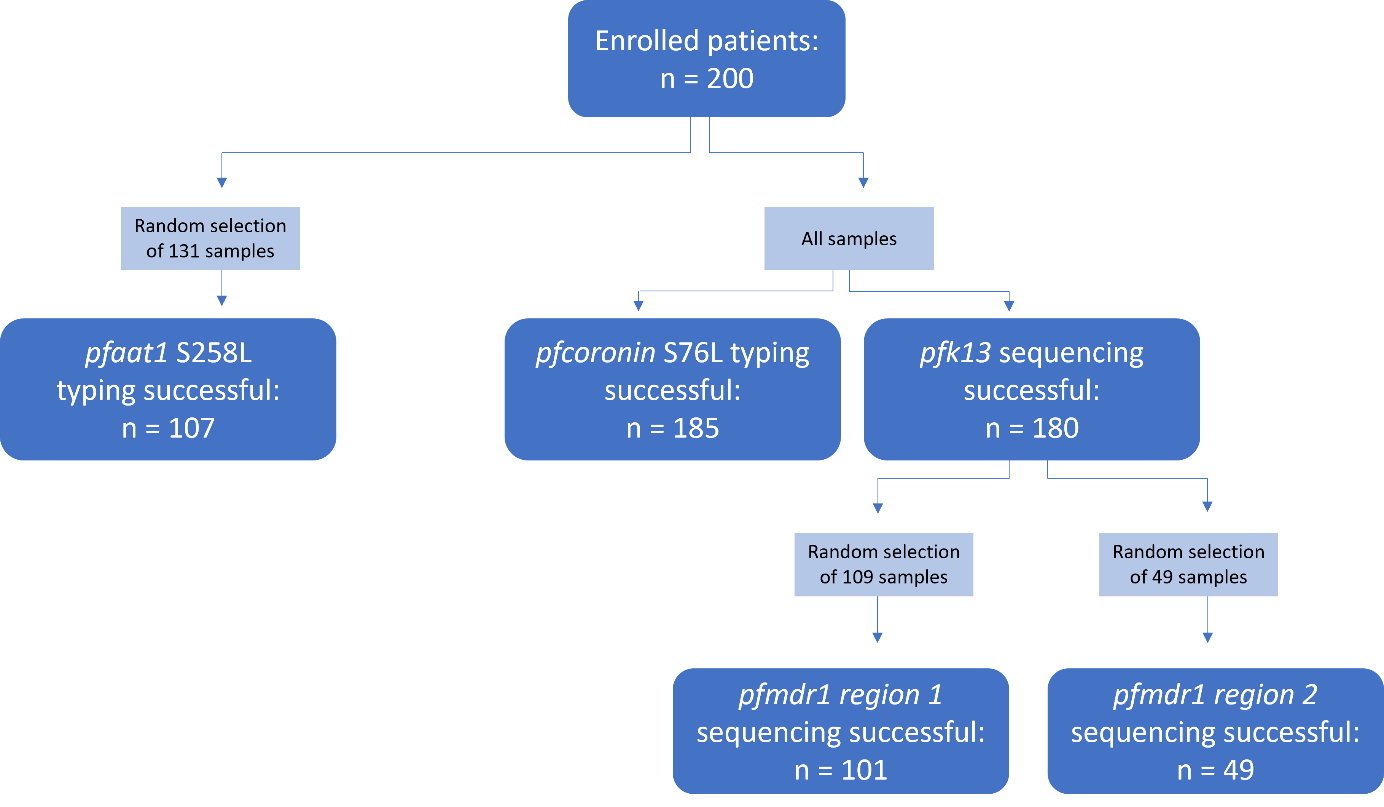
**
